# Identification of a Cannabinoid Receptor 2 Allosteric Site Using Computational Modeling and Pharmacological Analysis

**DOI:** 10.1021/acsptsci.4c00547

**Published:** 2025-01-28

**Authors:** Zara Farooq, Pietro Delre, Stylianos Iliadis, Giuseppe Felice Mangiatordi, Marialessandra Contino, Lesley A. Howell, Peter J. McCormick

**Affiliations:** † Centre for Endocrinology, William Harvey Research Institute, Bart’s and The London School of Medicine and Dentistry, Queen Mary University of London, Charterhouse Square, London EC1M 6BQ, U.K.; ‡ CNR-Institute of Crystallography, Via Amendola 122/o, Bari 70126, Italy; § Department of Pharmacy-Drug Sciences, University of Bari Aldo Moro, Via Orabona 4, Bari 70125, Italy; ∥ School of Physical and Chemical Sciences, Queen Mary University of London, Mile End Road, London E1 4NS, U.K.; ⊥ Department of Pharmacology and Therapeutics, Institute of Systems Integrative and Molecular Biology, University of Liverpool, Liverpool L69 7BE, U.K.; # XJTLU-University of Liverpool Joint Centre for Pharmacology and Therapeutics, Liverpool L69 7ZX, U.K.

**Keywords:** cannabinoid receptor 2 (CB_2_), G protein-coupled receptor (GPCR), allosteric, computational, signaling

## Abstract

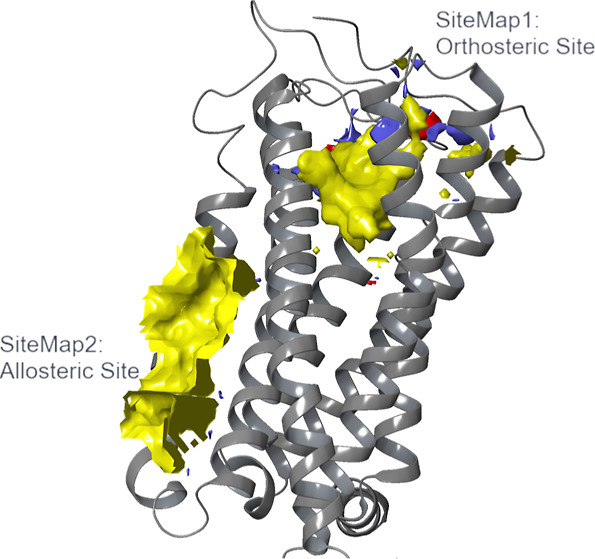

Emerging evidence has demonstrated that cannabinoid receptor 2 (CB_2_) is involved in a number of diseases, such as neurodegenerative disorders and various types of cancer, making it an attractive pharmacological target. Classically, a protein active site or an orthosteric binding site, where the endogenous ligand binds to, is used as a target for the design of most small-molecule drugs. This can present challenges when it comes to phylogenetically related proteins that have similar orthosteric binding sites, such as the cannabinoid receptors. An alternative approach is to target sites that are unique to these receptors yet still impact receptor function, known as allosteric binding sites. Using an inactive-state human cannabinoid receptor 2 crystal structure (PDB ID:5ZTY), we identified a putative CB_2_ allosteric site using computational approaches. In vitro signaling assays using known allosteric modulators and CB_2_ agonists have been used to verify the in silico results. This identification opens promising avenues for the development of selective and specific CB_2_ ligands for therapeutic purposes.

G protein-coupled receptors (GPCRs) make up the largest class of membrane proteins consisting of around 800 members.^1^ Also termed the 7-transmembrane (TM) receptors, GPCRs are located on the cell membrane and transduce extracellular signals into important physiological effects such as regulation of immune system activity, behavioral management, as well as being involved in growth and metastasis, making GPCRs attractive pharmacological targets.^2^ Class A GPCRs, also known as rhodopsin-like receptors, are the largest class of the GPCR superfamily, and given their wide range of physiological functions, they are also the most therapeutically targeted among other classes.^3^ Cannabinoid receptor 1 (CB_1_) and cannabinoid receptor 2 (CB_2_) are two important class A GPCRs and part of the endocannabinoid system which is responsible for the regulation of many biological processes in the body.^4^ The cannabinoid receptors (CBs) are located throughout the body with CB_1_ mainly expressed in the central nervous system and CB_2_ mostly found in the peripheral nervous system and immune system except in pathological settings.^5,6^ The CBs are linked to many diseases, with CB_2_ associated with neurodegenerative diseases, inflammation, and various cancer types such as colon, brain, liver, lung, blood, and breast, making CB_2_ an appealing drug target.^7,8^

Classically, a protein active site or orthosteric binding site of a GPCR is where the endogenous ligand binds and is generally used as a target for the design of most small-molecule drugs.^9^ Although this approach has shown to be effective in many cases, achieving selectivity among phylogenetically related GPCRs via targeting their orthosteric binding site can be a complex task.^10,11^ For example, CB_1_ and CB_2_ share 44% sequence identity, which increases to 68% when considering the transmembrane residues alone.^12^ Allosteric sites are situated at topographically distinct locations to that of the orthosteric binding sites; therefore, targeting these alternative sites can offer a solution to develop selective and specific therapeutics for structurally similar proteins.^13^ Upon binding, allosteric ligands, also known as allosteric modulators (AMs), are able to regulate signal transduction pathways as well as conformational changes, providing novel strategies for developing therapeutics for GPCRs.^2,14^ Moreover, allosteric sites tend to be structurally less conserved due to low evolutionary selection pressure, even between receptor subfamily members with high homology such as CB_1_ and CB_2_, allowing for the opportunity for selective receptor binding.^15^ AMs can enhance or inhibit the agonist affinity, efficacy, and binding of orthosteric ligands; these are known as positive allosteric modulators (PAMs) and negative allosteric modulators (NAMs).^16^ AMs usually lack intrinsic activity and therefore are less likely to produce side effects due to their dependency on orthosteric ligands.^17^ When it comes to the CBs, this becomes useful as targeting allosteric sites can aid in reducing notorious off-target side effects that orthosteric ligands exhibit, such as depressant and psychomimetic effects.^18,19^ Due to the increase in interest and potential significance, there has been a number of allosteric sites identified among class A GPCRs located in areas such as interior and lipid-facing exterior of the TM helix bundles as well as intracellular and extracellular regions, including that of muscarinic acetylcholine receptor 2 (M_2_), beta-2 adrenergic receptor (β_2_AR), chemokine receptor 5 (CCR5), and adenosine A_2A_ receptor (A_2A_R), all of which have cocrystallized structures with allosteric ligands bound.^20^ The allosteric sites located at the lipid interface embedded in the cell membrane are the most unusual, which was demonstrated when the X-ray crystal structure of the human CB_1_ receptor in complex with NAM, ORG27569, and agonist CP 55,940 was established (PDB ID: 6KQI).^21^ This structure showed that the CB_1_ allosteric site is located at the extrahelical site in the inner leaflet of the membrane, also known as LOW34.^22^ UP34, UP12, LOW67, and LOW345, which are the four locations identified by crystal structures demonstrating unusual locations, where UP refers to the extracellular end (i.e., the upper part) and LOW refers to the cytoplasmic end (i.e., the lower part) and the numbers refer to the main interacting TM helices.^22^

Ec2la is the first reported synthetic small-molecule AM of CB_2_, which is one of the few reported CB_2_ AMs.^16,23^ In the literature, Ec2la was mentioned to be a PAM for CB_1_ and CB_2_ receptors in [^3^H]CP 55,940 binding studies in Chinese hamster ovary cells.^23^ The capability of the two agonists, CP 55,940 and 2-arachidonoylglycerol (2-AG), to stimulate [^35^S]GTPγS binding was greatly enhanced by Ec2la for CB_2_, once more supporting its PAM modality in these experiments.^23^ As a result, Ec2la was chosen to be tested in subsequent experiments in order to aid the identification of an CB_2_ allosteric site. Cannabidiol (CBD) has been reported to act as an antagonist, agonist, partial agonist, and NAM for the CB_2_ receptor,^24−27^ as well as an antagonist, agonist, and NAM for the CB_1_ receptor.^9,27−29^ In the literature, the actual CBD modality is controversial when it comes to its interactions with CB_1_ and CB_2_.^30^ Consequently, CBD was included in our cell signaling experiments to examine its potential role as an AM for CB_2_. The structural determination of human CB_2_ via X-ray crystallography (PDB ID: 5ZTY)^31^ has enabled the prospective discovery of a CB_2_ allosteric site using in silico methods. The generated results were verified using in vitro signaling assays using the known allosteric modulator, Ec2la, and CB_2_ agonists. The present identification and validation of a putative CB_2_ allosteric binding site provides promising opportunities for the development of selective CB_2_ ligands targeting many diseases.

## Results and Discussion

### Computational Identification of a Putative CB_2_ Allosteric Site

For the prediction of putative allosteric binding sites on CB_2_, the SiteMap cavity finding algorithm^32−34^ was implemented on the inactive-state human CB_2_ crystal structure (PDB ID: 5ZTY).^31^ SiteMap^32−34^ identified five cavities, three of which had values below the indicated SiteScore and Dscore thresholds and are not discussed and investigated further. The top two identified SiteMaps based on SiteScore are listed in (Table 1), along with their SiteScore and Dscore values. The former serves to identify protein cavities that would be suitable as drug-binding sites, while the latter is used for evaluating their druggability. The orthosteric binding site with the cocrystallized ligand, AM10257, was correctly identified and scored with the highest SiteScore, as it overlapped with SiteMap1 (SM1). The second identified binding site, SiteMap2 (SM2), was detected at an extrahelical location and predicted to have a SiteScore value above 1, which sets it as a promising binding site. Druggability assessment of the two sites, as evaluated from SiteMap’s Dscore metric, suggested that both of them are druggable, since they possess values greater than or very close to 0.98.^34^

**Table 1:** SiteScore and Dscore Values for the Two Identified Druggable Cavities from SiteMap^a^

CB_2_ SiteMap cavities	SiteScore	Dscore
SiteMap1 (SM1)	1.258	1.329
SiteMap2 (SM2)	1.110	1.216

a SiteScore and Dscore values for the top two identified cavities returned from SiteMap based on SiteScore are listed in descending order. SiteScore indicates the potential of an identified cavity as a drug-binding site and serves as binding site identification. Predicted cavities with SiteScore > 1 are likely to be drug-binding sites, whereas values less than 0.80 are considered “undruggable” sites.^34^ SiteScore does not account for binding site druggability evaluation, for which the Dscore metric is used. Sites with Dscore > 0.98 are considered as druggable.^34^ Given these definitions, both identified cavities are potential druggable binding sites. SM1 overlaps with the orthosteric binding site where the cocrystallized ligand, AM10257, was bound, whereas SM2 is a potential CB_2_ allosteric site at an extrahelical location predominantly between TM3, TM4, and TM5.

**Figure 1:**
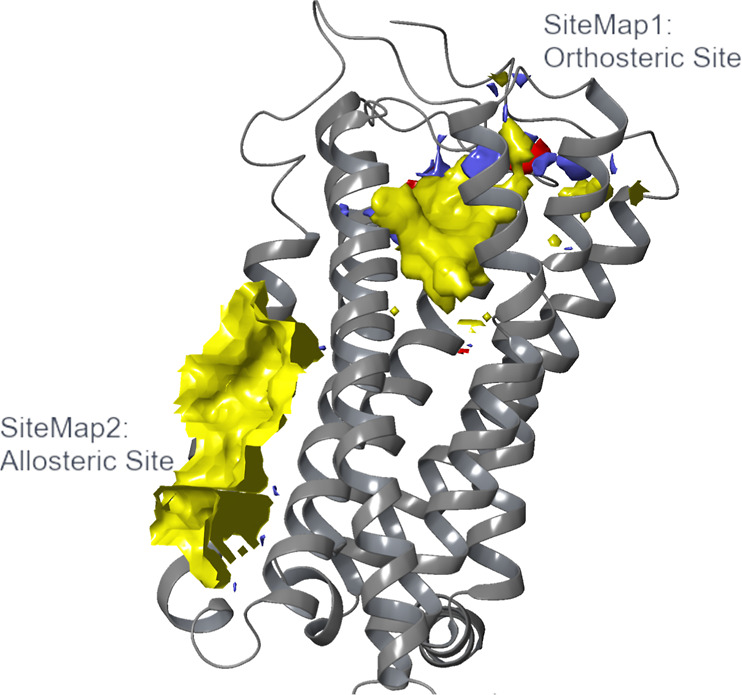
Visual surface of the putative CB_2_ allosteric site. SiteMap1 overlaps with the orthosteric binding site where the co-crystalized ligand, AM10257, was bound (PDB ID:5ZTY).^31^ SiteMap2 is a putative extrahelical allosteric pocket on the CB_2_ receptor with predominant hydrophobic properties, as demonstrated by the yellow surface. The figure was generated in Maestro.^35^

The two predicted binding sites had different locations in the GPCR transmembrane domain, which gives rise to their distinct properties. While SM1 was found in the orthosteric binding site at the extracellular-facing TM cavity, SM2 was found to be located mainly across TM3, TM4, and TM5 as an extrahelical binding site. As SM2 is positioned at the membrane–protein interface, it is highly hydrophobic in nature indicated by the yellow hydrophobic site map in this region (Figure 1). In contrast, SM1’s properties are more balanced, with hydrophobic, hydrogen-bond donor (blue surface), and hydrogen-bond acceptor binding properties (red surface) present. The absence of the latter two properties in SM2 highlights the different qualities of the two identified sites, and therefore, the potential differences between ligands that could bind to them. Based on these results, SM2 was carried forward for further analysis as a putative allosteric pocket on the CB_2_ receptor.

Importantly, SiteMap was also run on the active-state X-ray crystal structure of the human CB_2_ receptor in complex with agonist, AM12033, (PDB ID: 6KPC),^36^ in order to further verify our initial findings. The software identified exactly the same allosteric cavity (SM2), with Dscore and SiteScore values comparable to those obtained using the inactive-state X-ray crystal structure (PDB ID: 5ZTY)^31^ (Dscore: 1.020 vs 1.216 and SiteScore: 0.951 vs 1.110). This finding is consistent with the high degree of similarity between the two structures, as evidenced by the low root-mean-square deviation (RMSD) value obtained after alignment (0.997 Å). It is also worth noting that an even lower value is obtained when the RMSD is calculated for the identified allosteric cavity alone (0.440 Å), demonstrating that the two structures (within the scope of this study) do not appear significantly different. These results suggest that SiteMap is a useful and reliable tool to identify allosteric sites within receptors, whether the structure used is an active- or inactive-state model.

### Comparison of CB_1_ and CB_2_ Allosteric Sites

**Figure 2:**
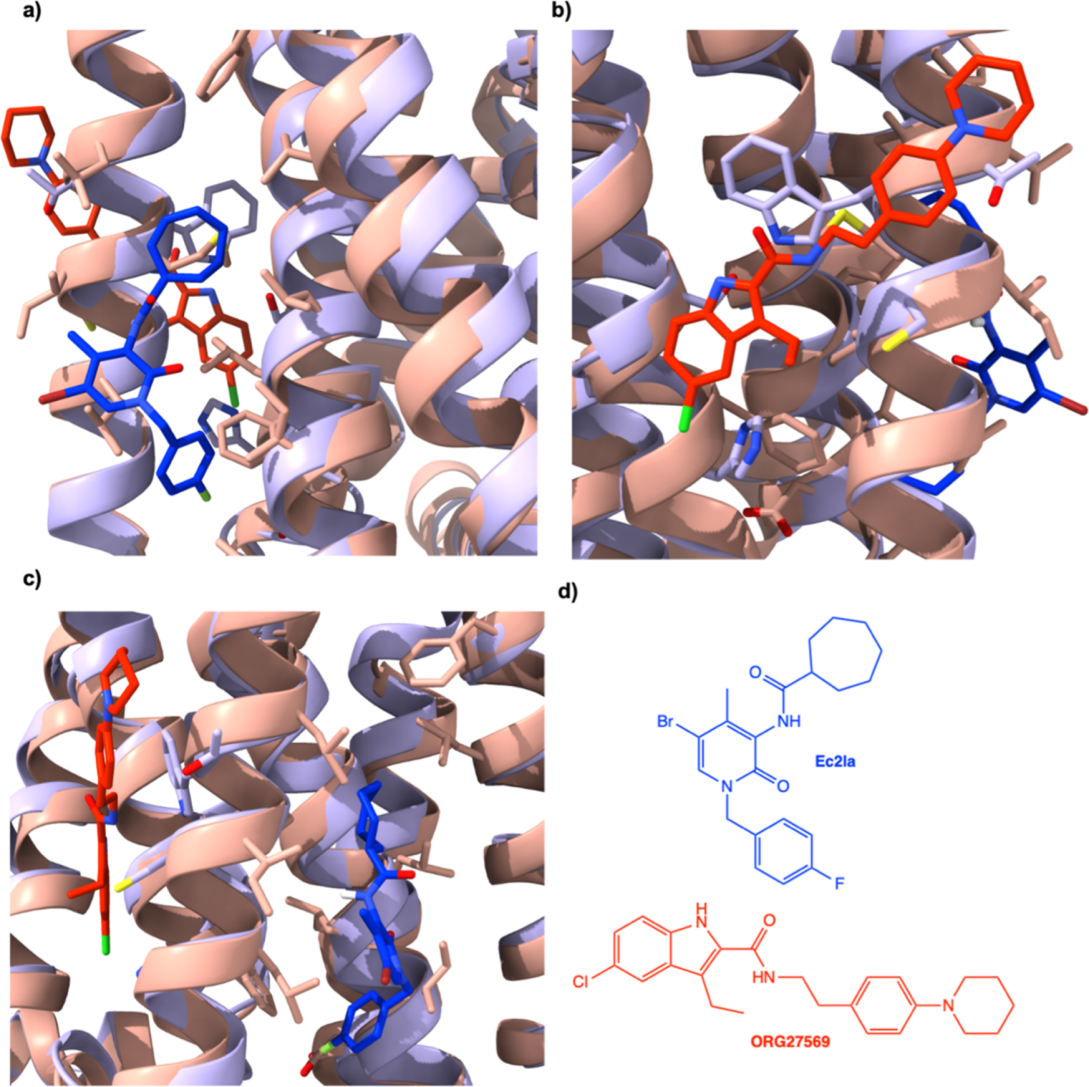
Comparison between the CB_1_ allosteric binding site (PDB ID: 6KQI)^21^ and the putative CB_2_ allosteric site (PDB ID: 5ZTY).^31^ Structural alignment of CB_1_^21^ (lilac) with allosteric modulator, ORG27569 (red), bound and CB_2_ (salmon-pink) with allosteric modulator, Ec2la (blue), bound. (a) View of Ec2la (blue) in CB_2_ (salmon-pink) binding pocket. (b) View of ORG27569 (red) in CB_1_^21^ (lilac) binding pocket. (c) Side view of both allosteric modulators, Ec2la (blue) and ORG27569 (red), in their corresponding allosteric sites CB_1_^21^ (lilac) and CB_2_ (salmon-pink), respectively. (d) Structures of Ec2la and ORG27569. Alignment demonstrates that both allosteric sites are found in similar protein portions of each receptor. Although they are in similar positions, they are not overlapping; therefore, the sites are significantly different, increasing the chances of designing specific CB_2_ allosteric modulators. The CB_1_^21^ complex is an experimental structure solved with the allosteric modulator, ORG27569, bound and the CB_2_ structure bound to allosteric modulator, Ec2la, is from docking studies. The experimental structure for CB_2_ used is from the inactive-state human X-ray crystal structure.^31^ Images were generated using UCSF ChimeraX.^38^

Shao and colleagues identified an allosteric site within the CB_1_ receptor by solving the structure of ORG27569, a NAM bound to an extrahelical site that is situated within the inner leaflet of the membrane.^21^ Due to the structural similarities between CB_1_ and CB_2_, this inactive-state crystal structure (PDB ID: 6KQI) was imported and aligned with the CB_2_ inactive-state crystal structure to detect whether the possible allosteric binding sites for each protein were in the same protein portion. It was found that the two binding sites (for CB_1_ demonstrated by Shao et al., for CB_2_ predicted by SiteMap, SM2) are close to each other (Figure 2). Although in close proximity, the two sites do not overlap; therefore, the sites are distinct. Moreover, none of the other sites predicted by SiteMap were located in the same region as the CB_1_ allosteric site; SM2 is the closest among the predicted sites to the CB_1_ allosteric site. Recent studies demonstrated by Shen and colleagues further confirms that the CB_1_ and CB_2_ allosteric sites are different by a CB_1_ allosteric ligand, CB-05, elucidating selectivity toward the CB_1_ receptor over the CB_2_ receptor.^37^ These conclusions, coupled with recent studies, strengthen not only the findings that SM2 is a good prediction for a CB_2_ allosteric site but also that the CB_1_ and CB_2_ allosteric sites are in different locations, increasing chances of specificity.

### Molecular Docking of CB_2_ Allosteric Ligands in SM2 to Identify Ligand Binding Residues

**Figure 3:**
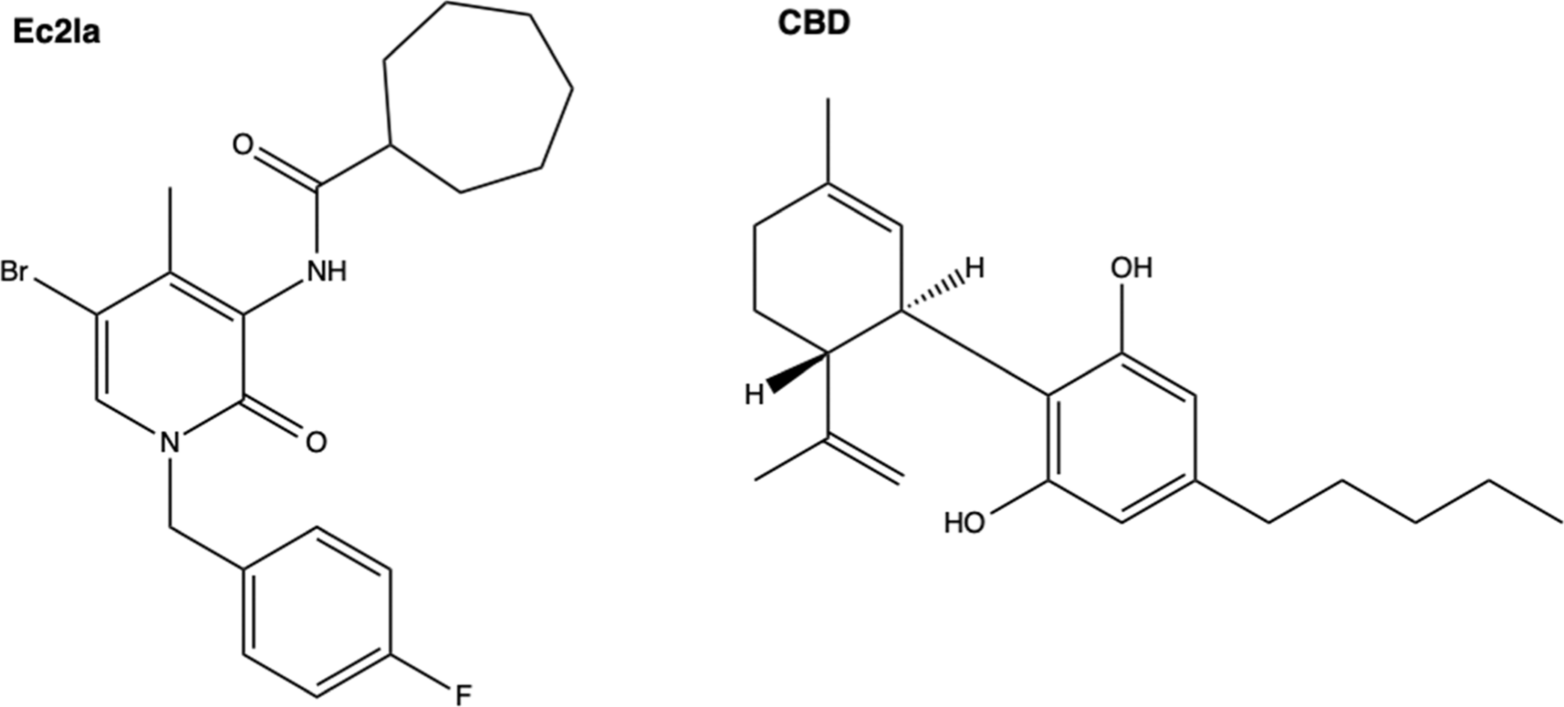
Chemical structures of reported small-molecule CB_2_ allosteric modulators, Ec2la and CBD.

**Figure 4:**
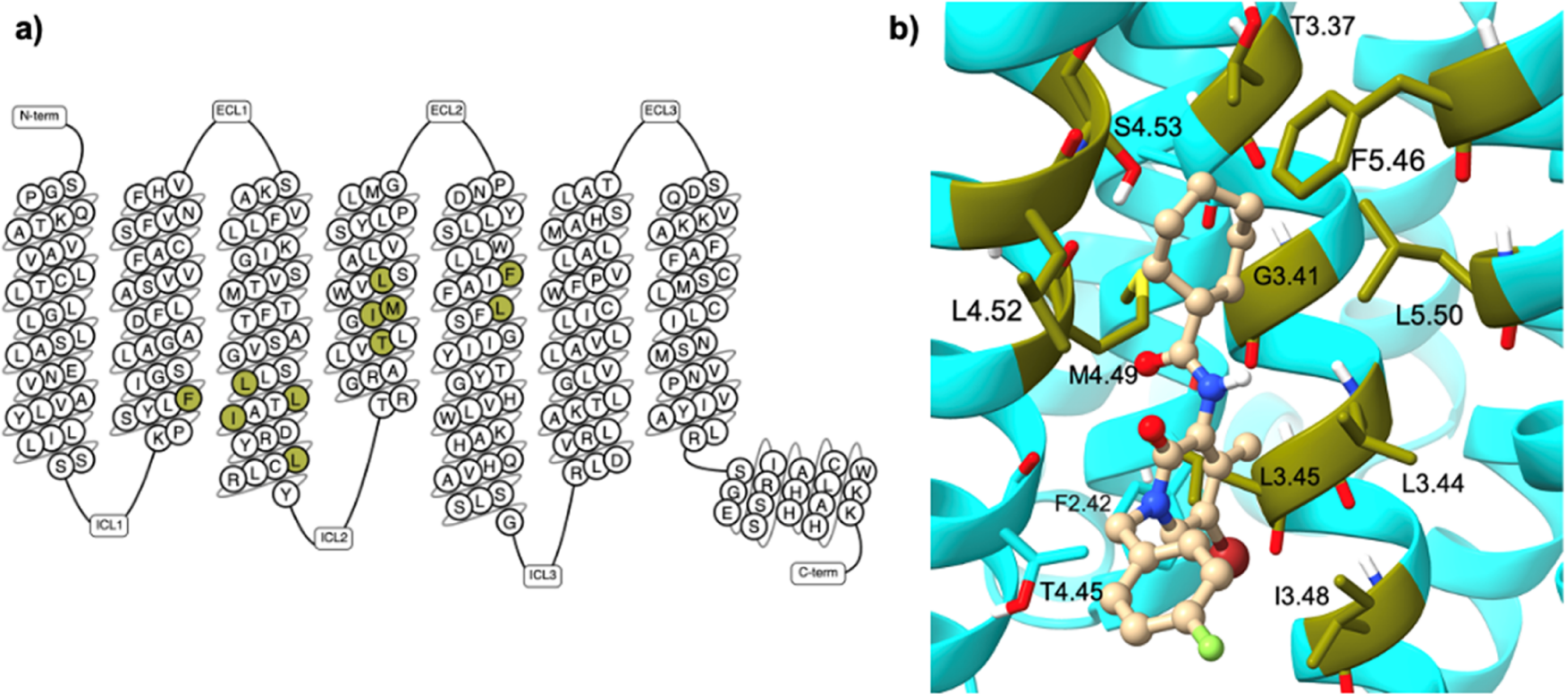
Amino acids residues within the putative allosteric pocket showing interactions between the small-molecule ligand, Ec2la, and the CB_2_ receptor. (a) Snake-plot of CB_2_ demonstrating the location of the 11 residues constituting the mutation set interacting with Ec2la, generated using GPCRdb tools.^42^ (b) Top-scored docking pose of Ec2la (in ball-and-stick representation in beige) in SM2 with the ligand-binding residues shown in olive. CB_2_ is shown in cyan. Image was generated by UCSF ChimeraX.^38^

Following the prediction of SM2 as a potential CB_2_ allosteric binding site, molecular docking was performed to identify the ligand-binding residues involved in this putative site. Known small-molecule CB_2_ allosteric ligands, Ec2la and CBD,^16^ (Figure 3) were then prepared and docked onto SM2. After the docking calculations were completed, the generated poses for each ligand were reviewed based on docking scores (Table S1) and visual inspection, and all interactions between the top-scored pose of each ligand and the receptor (Figure S1) were established (Table S2). Not surprisingly, due to the nature of the pocket, hydrophobic interactions were the predominant observed interaction type. Ec2la is the first published synthetic small-molecule allosteric modulator for CB_2_, therefore the interactions between Ec2la and CB_2_ were studied for subsequent experimental analysis.^23^ The ligand–receptor contacts of the top-scored pose of Ec2la indicated 11 amino acids, located predominantly at TM3, TM4, and TM5 (Figure 4) to form the proposed allosteric pocket. Guided by this, the following residues were chosen to be studied experimentally: F72^2.42^, L125^3.44^, L126^3.45^, I129^3.48^, L133^3.52^, T153^4.45^, I156^4.48^, M157^4.49^, L160^4.52^, F197^5.46^, and L201^5.50^ (the Ballesteros–Weinstein generic residues numbering scheme is used).^39^ As a result, these 11 amino acids were individually mutated to alanine during site-directed mutagenesis studies to assess their importance within this proposed binding pocket.

Yuan et al. published in silico predicted allosteric binding sites within the CB_2_ receptor with sites in similar locations to SM2.^46^ Among these sites, sites B and K overlap with SM2. Moreover, Gado et al. also recently published bitopic ligands binding to the orthosteric and a proposed allosteric binding site on the CB_2_ receptor.^41^ The allosteric part of the bitopic ligand was predicted to bind to an allosteric site in a location similar to that of SM2. These results further solidify our findings that SM2 is a good prediction for a putative allosteric binding site within the CB_2_ receptor.

### Verification of the Putative Allosteric Binding Site through Signaling Experiments

**Figure 5:**
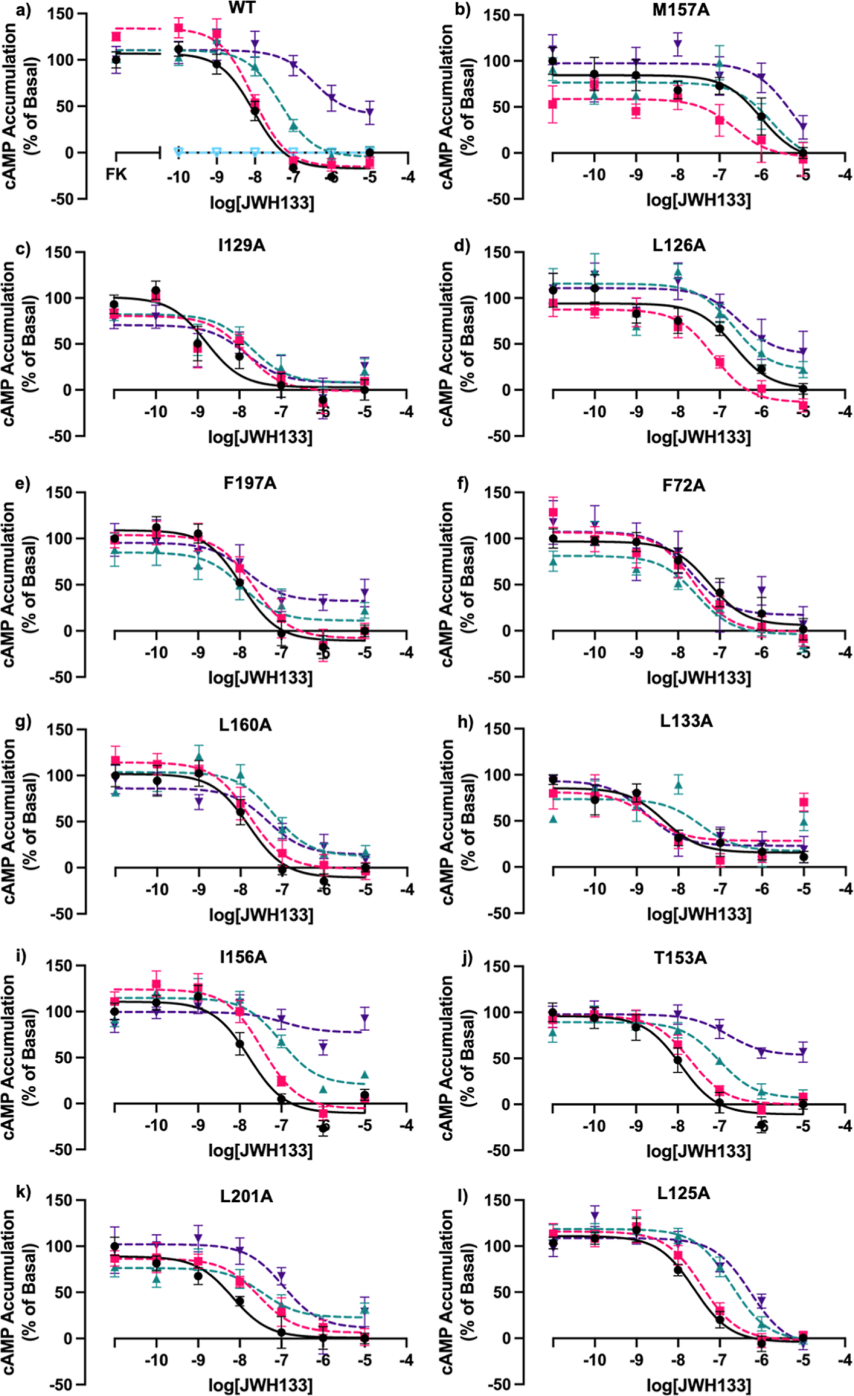
Effect of Ec2la on WT CB_2_ and CB_2_ mutants in the presence of CB_2_ agonist JWH133 in cAMP accumulation assays. FK-induced (7.5 μM) cAMP accumulation dose–response curves monitoring the effect of Ec2la on WT CB_2_ and CB_2_ mutants using a CB_2_ selective agonist, JWH133. (a) WT CB_2_, (b) M157^4.49^A, (c) I129^3.48^A, (d) L126^3.45^A, (e) F197^5.46^A, (f) F72^2.42^A, (g) L160^4.52^A, (h) L133^3.52^A, (i) I156^4.48^A, (j) T153^4.45^A, (k) L201^5.50^A, and (l) L125^3.44^A. Figure 3b–h shows cAMP signaling curves of CB_2_ mutants demonstrating a loss and/or alteration in allosteric signal in the presence of CB_2_ allosteric modulator, Ec2la, and CB_2_ selective agonist, JWH133. Figure 3i–l shows cAMP signaling curves of CB_2_ mutants still demonstrating an allosteric effect in the presence of CB_2_ allosteric modulator, Ec2la, and CB_2_ selective agonist, JWH133. Although these mutants still remain to have an allosteric effect, there is still an alteration of the signal demonstrating that these amino acids could be involved in the proposed site, but it is less likely. [Black = vehicle control (no Ec2la), pink = 0.1 μM Ec2la, green = 1 μM Ec2la, purple = 10 μM Ec2la, light blue = Ec2la alone]. FK alone represents 100%. Data are represented as mean ± SEM as percentage of accumulation normalized to the vehicle control from three independent experiments done in triplicate. [See Table S4 for pEC_50_/pIC_50_ values where statistical tests were performed to compare the pIC_50_/pEC_50_ values of each condition of Ec2la (0.1, 1, 10 μM) vs vehicle control (no Ec2la) in GraphPad Prism using a repeated measures one-way ANOVA with Dunnett’s multiple comparisons test (* < 0.05)].

CB_2_ couples to Gα_*i*_ and therefore leads to a downstream effect of inhibition of cyclic adenosine monophosphate (cAMP) production via adenylate cyclase. CB_2_ was transiently transfected into human embryonic kidney 293 (HEK293) cells and treated with Ec2la to confirm its allosteric activity, while titrating in a dose–response of JWH133 (*K*_*i*_ = 3.4 nM)^43^ in a cAMP signaling assay, using the GloSensor biosensor. JWH133 was used as the primary agonist in subsequent studies due to its high selectivity and potency toward CB_2_. Ec2la was tested at three different concentrations (0.1, 1, 10 μM) on WT CB_2_ in the presence of JWH133, with 10 μM showing the most significant allosteric shift. It was shown that Ec2la acts like a NAM for CB_2_ in the presence of JWH133 due to the inhibition of cAMP signaling; the potency and efficacy are decreased which can be observed by the shift in EC_50_ and the *E*_MAX_ compared to that of the vehicle control, and the curve being shifted rightward (Figure 5a). Following, we tested the ubiquity of Ec2la’s allosteric activity by testing it in the presence of the CB_2_-selective agonist HU308, the nonselective cannabinoid receptor agonist CP 55,940, endogenous cannabinoid ligand 2-AG, as well as CBD. In the presence of CP 55,940 and 2-AG, Ec2la showed a negative allosteric shift at 10 μM, whereas at nanomolar concentrations, Ec2la had no allosteric effect (Figures S2 and S3). Interestingly, in the presence of agonist HU308, Ec2la acts as a PAM, demonstrating the significance of probe dependence in relation to allosteric modulation and the importance of the interaction and cooperativity between the orthosteric and allosteric ligands. This phenomenon of biased modulation is also seen with the structurally similar CB_1_ receptor and its allosteric ligand, ORG27569, where its distinctive function can be demonstrated through its positive allosteric effect in ERK 1/2 signaling experiments yet a negative allosteric effect with radioligand binding in the presence of orthosteric ligand CP 55,940.^44^ Its interesting function and PAM-antagonistic effect on CB_1_ has sparked interest in the functional behavior of AMs linked to probe dependence and biased agonism or modulation.^45^ This research is important in relation to the CBs as it is apparent that from our results, Ec2la could also have a similar distinctive profile, demonstrating its probe dependence with specific agonists, but also its positive allosteric effect in binding assays, as previous studies have suggested.^23^

All 11 mutants were then tested under the same conditions as WT CB_2_, resulting in M157^4.49^A, I129^3.48^A, L126^3.45^A, F197^5.46^A, F72^2.42^ A, L160^4.52^A, and L133^3.52^A, demonstrating a loss or alteration in allosteric signal (Figure 5b–h), suggesting their involvement in the proposed binding site. Mutants I156^4.48^A and T153^4.45^A still demonstrate a shift in EC_50_ and the *E*_MAX_, similar to that of WT CB_2_ (Figure 5i,j). Although mutants L201^5.50^A and L125^3.44^A still demonstrate a shift in EC_50_, they do not exhibit a change in *E*_MAX_ shown by WT CB_2_ (Figure 5k,l). Due to the signal being altered, it could suggest that they may be involved in the putative site, but it is not as likely as the other amino acids that have a definite change or loss in the allosteric signal. As a result, these data show that 7 out of the 11 possible amino acids altered allosteric activity, demonstrating that they are likely to be involved in this putative allosteric pocket.

**Figure 6:**
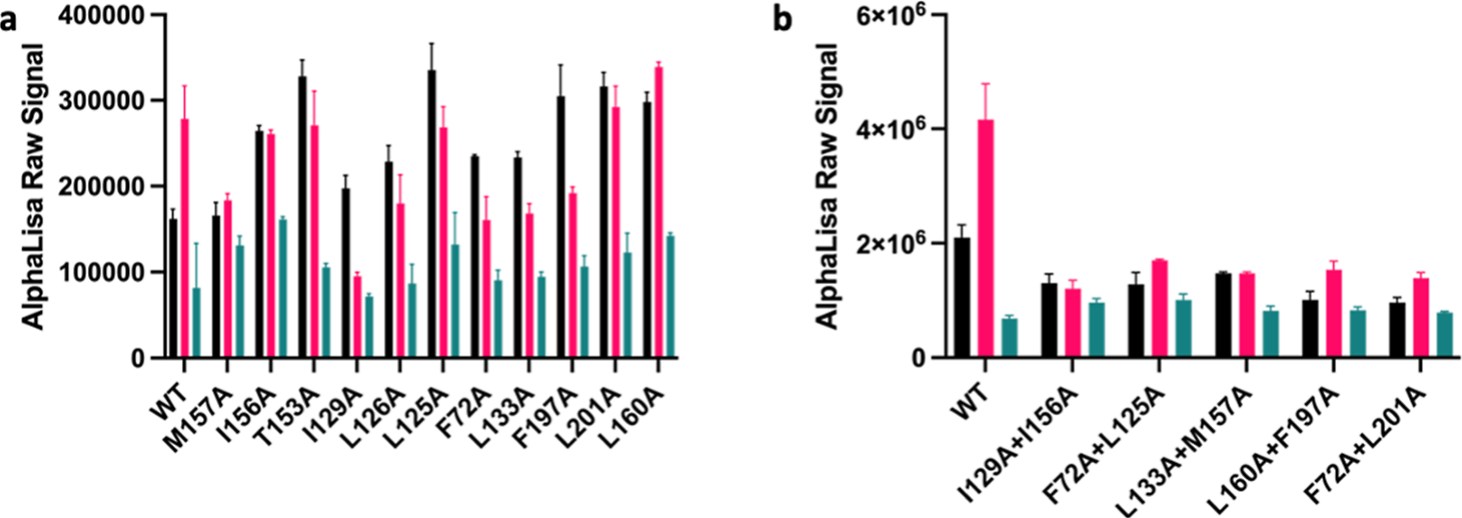
Effect of Ec2la on CB_2_ in the presence of CB_2_ agonist JWH133 on WT CB_2_ and CB_2_ mutants in *p*-ERK 1/2 signaling assay. (a) *p*-ERK 1/2 signaling of WT CB_2_ and CB_2_ single mutants in the presence of 1 μM JWH133 + 10 μM Ec2la (black), 1 μM JWH133 alone (pink), and 10 μM Ec2la alone (green); (b) *p*-ERK 1/2 signaling of WT CB_2_ and CB_2_ double mutants in the presence of 1 μM JWH133 + 10 μM Ec2la (black), 1 μM JWH133 alone (pink), and 10 μM Ec2la alone (green). For WT CB_2_, Ec2la acted as a NAM in *p*-ERK 1/2 signaling. For the CB_2_ mutants, there was either no effect or an increase in the *p*-ERK 1/2 signal. When the CB_2_ receptor had a double mutation, allosteric effects were lost with some double mutants, while the receptor still retained activity. Data are represented ± SEM from three independent experiments done in duplicate.

Our data suggest that Ec2la inhibits cAMP production and therefore acts as a NAM. These findings contrast with studies that suggest that Ec2la acts as a PAM in binding studies^23^ and inspired the investigation of alternative pathways such as its downstream signaling of phosphorylated-extracellular signal-related kinase *p*-ERK 1/2. Ec2la reduced the amount of *p*-ERK 1/2 when in the presence of JWH133 with WT CB_2_, demonstrating that Ec2la also acts as a NAM in *p*-ERK 1/2 signaling (Figure 6a). Interestingly, Ec2la did not show a negative allosteric effect for the CB_2_ mutants; instead, there was either no effect or an increase in the *p*-ERK 1/2 signal. Double mutations were then made to further validate these findings; I129^3.48^A + I156^4.48^A, F72^2.42^A + L125^3.44^A, L133^3.52^A + M157^4.49^A, L160^4.52^A + F197^5.46^A, and F72^2.42^A + L201^5.50^A. These mutants were specifically designed as each single mutation in every double mutation pair could be found on different transmembrane regions on the receptor. It was found that when the CB_2_ receptor had a double mutation, there was either no effect to the allosteric modulator-induced changes on the signal or a slight decrease in the signal, while the receptor still retained activity (Figure 6b). These results suggest that with the double mutants, Ec2la does not possess the same negative allosteric properties compared with WT. These findings do not directly correspond to that of the cAMP data; however, considering *p*-ERK 1/2 activation may be arrestin-dependent or occurring at a later time point than cAMP, it is possible to observe different effects of the ligand towards the receptor. However, we can see for both single and double mutants that the effect that Ec2la has in the presence of JWH133 compared to that of WT is conclusively distinct, implicating the proposed site as the allosteric site for Ec2la.

Protein expression levels of WT CB_2_ and CB_2_ mutants (F72^2.42^A, L125^3.44^A, L126^3.45^A, I129^3.48^A, L133^3.52^A, T153^4.45^A, I156^4.48^A, M157^4.49^A, L160^4.52^A, F197^5.46^A, and L201^5.50^A) were initially tested using western blot, demonstrating that all 12 CB_2_ variants had appropriate protein expression levels. L201^5.50^A and I156^4.48^A showed a reduced expression by western blot (Figure S4a), and they also remained to have allosteric activity in the presence of Ec2la in cAMP experiments. An in-cell western was also performed to further confirm these findings that protein expression is similar throughout all of the mutants. The results demonstrated that there was no significant difference between the WT-permeabilized cells compared to the mutant permeabilized cells and mutant nonpermeabilized cells, apart from M157^4.49^A, I156^4.48^A, and L133^3.52^A permeabilized cells (Figure S4b). However, there was no significant difference when the cells were not permeabilized, which indicates there is not a problem with trafficking or translation.

cAMP data demonstrate that our in silico binding site is verified; all but 4 of the mutated residues were shown to be involved in the allosteric binding pocket. It was found that Ec2la acted as a NAM at 1 and 10 μM with WT CB_2_ in the presence of the CB_2_-selective agonist, JWH133 (Figure 5a). Consequently, with the CB_2_ mutants, we expected to observe a shift in this negative allosteric signal compared with WT. In fact, mutations M157^4.49^A, I129^3.48^A, L126^3.45^A, F197^5.46^A, F72^2.42^ A, L160^4.52^A, and L133^3.52^A showed a loss in the cAMP signal under the same conditions as WT. These data confirm our in silico findings and the location of the putative allosteric binding site. Our site was also recently found to be located in a similar location demonstrated by Yuan et al. with their in silico prediction of CB_2_ allosteric binding sites.^46^ Sites B and K are shown to have some of the same amino acid residues that were found during our in silico analysis with SiteMap. These findings further strengthen our discovery of this CB_2_ allosteric site. Conversely, their findings suggest that site H, located very close to the orthosteric binding site, is their reported most promising site; interestingly, the location of this site was not detected during our search for putative sites using SiteMap. Upon performing *p*-ERK 1/2 assays to investigate an alternative pathway involved in the signaling of the CB_2_ receptor, it was found that Ec2la also had a negative allosteric effect on WT CB_2_ (Figure 6a). Ec2la reduces the *p*-ERK 1/2 signal in the presence of JWH133, similarly to cAMP experiments. However, the data are not comparable with the single mutants; Ec2la does not seem to have the same effect as WT CB_2_ and in some cases even had an opposing effect. Double mutations were then prepared to examine whether the effects on these mutants with the *p*-ERK 1/2 signal were different. The double mutations did not show the same large reduction in the signal as WT CB_2_ did in the presence of Ec2la and JWH133, as well as having different effects than the single mutants. These findings, particularly from the double mutants, were able to further support the validity of our putative allosteric binding site.

### Cannabidiol Behaves as a NAM

**Figure 7:**
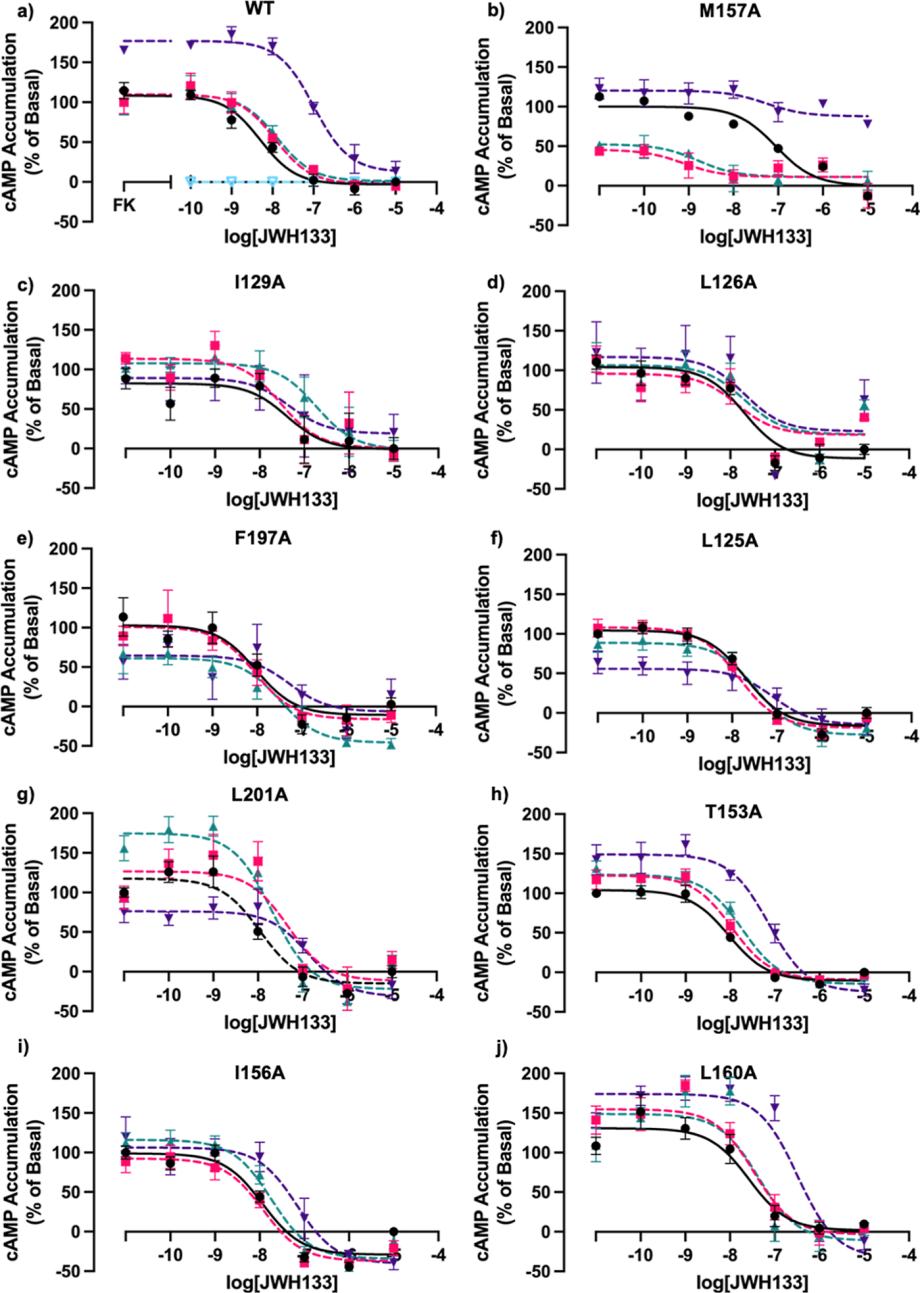
Effect of CBD on CB_2_ in the presence of CB_2_ agonist JWH133 in cAMP accumulation assays. FK-induced (7.5 μM) cAMP accumulation inhibition dose–response curves monitoring the effect of CBD on WT CB_2_ and CB_2_ mutants using a CB_2_ selective agonist, JWH133. (a) WT CB_2_, (b) M157^4.49^A, (c) I129^3.48^A, (d) L126^3.45^A, (e) F197^5.46^A, (f) L125^3.44^A, (g) L201^5.50^A, (h) T153^4.45^A, (i) I156^4.48^A, and (j) L160^4.52^A. Figure 3b–g shows cAMP signaling curves of CB_2_ mutants demonstrating a loss and/or alteration in the allosteric signal in the presence of CB_2_ allosteric modulator, CBD, and CB_2_ selective agonist, JWH133. Figure 3h–j shows cAMP signaling curves of CB_2_ mutants still demonstrating an allosteric effect in the presence of CB_2_ allosteric modulator, CBD, and CB_2_ selective agonist, JWH133. Although these mutants still remain to have an allosteric effect, there is still an alteration of the signal demonstrating that these amino acids could be involved in the proposed site, but it is less likely. [Black = vehicle control (no CBD), pink = 0.1 μM CBD, green = 1 μM CBD, purple = 10 μM CBD, and light blue = CBD alone]. FK alone represents 100%. Data are represented as mean ± SEM as percentage of accumulation normalized to the vehicle control from three independent experiments done in triplicate. [See Table S5 for pEC_50_/pIC_50_ values, where statistical tests were performed to compare the pIC_50_/pEC_50_ values of each condition of Ec2la (0.1, 1, 10 μM) vs vehicle control (no Ec2la) in GraphPad Prism using a repeated measures one-way ANOVA with Dunnett’s multiple comparisons test (* < 0.05)].

During molecular docking studies, it was found that the reported AM, CBD, made hydrophobic interactions with the following residues: L125^3.44^, L126^3.45^, I129^3.48^A, T153^4.45^, I156^4.48^, M157^4.49^, L160^4.52^, F197^5.46^, and L201^5.50^ (Table S2). Not surprisingly, some of these same amino acids were also involved in making interactions with Ec2la. CBD was subsequently tested as an allosteric modulator in the presence of JWH133 as the agonist for the following mutations: L125^3.44^A, L126^3.45^A, I129^3.48^A, T153^4.45^A, I156^4.48^A, M157^4.49^A, L160^4.52^A, F197^5.46^A, and L201^5.50^A. The same mutants that had a loss of allosteric effect with Ec2la also demonstrated this change with CBD (Figure 7); this correlates with the in silico data and further validates our computational findings. In line with the literature stating CBD as an orthosteric and allosteric ligand, we see CBD acts as a NAM with WT CB_2_ (Figure 7a), decreasing the EC_50_ and shifting the curve rightward and upward, while also acting as an agonist in the presence of allosteric modulator, Ec2la (Figure S2d).^30^

Computational studies demonstrated that most of the residues that were shown to interact with Ec2la also interacted with CBD. CBD demonstrated negative allosteric activity with the CB_2_ receptor in the presence of JWH133 (Figure 7a). Navarro et al. have demonstrated at nanomolar concentrations, CBD possesses allosteric ability for the CB_2_ receptor, prompting their design of PAMs and NAMs for CB_2_ derived from CBD.^25,30^ Together with our findings and current research, CBD was then tested with the 9 mutations to explore if our in silico predictions could be verified. It was found that mutated residues that demonstrated a loss or change of the allosteric signal for Ec2la overlapped with those for CBD, further verifying our computational analysis.

## Conclusions

The development of novel AMs for the CB_2_ receptor could hugely impact this field of GPCR drug discovery, as it would serve as an excellent demonstration that structurally similar proteins can be targeted selectively, not only reducing off-target side effects but also benefiting particular disease states that are specific to that receptor. The first step in achieving this is to identify a CB_2_ allosteric site. Here, we identify a CB_2_ allosteric site using SiteMap and functionally validate this computational discovery using mutagenesis studies and signaling experiments. Although the site situated at the lipid interface embedded in the membrane has not been reported for many other class A GPCRs, the X-ray crystal structure of the human CB_1_ receptor bound to AM, ORG27569, does reveal similar characteristics. The comparison of the two allosteric sites demonstrated that the sites do not overlap suggesting they are significantly different and provide promise in designing specific ligands for each receptor. Mutagenesis and signaling experiments verified our in silico findings with 7 out of the 11 mutated amino acids (M157^4.49^A, I129^3.48^A, L126^3.45^A, F197^5.46^A, F72^2.42^A, L160^4.52^A, and L133^3.52^A) that were shown to be interacting with Ec2la in the binding site demonstrated a loss or change in signal, and 6 out of 9 mutated amino acids (L125^3.44^A, L126^3.45^A, I129^3.48^A, I156^4.48^A, M157^4.49^A, and F197^5.46^A) that were shown to be interacting with CBD in the binding site demonstrated a loss or change in signal. These results confirmed the location of the computationally predicted putative CB_2_ allosteric binding site. To further verify these results, the mutants were tested in *p*-ERK 1/2 activation experiments. It was found that although Ec2la demonstrated a reduction in *p*-ERK 1/2 signaling at the WT CB_2_ receptor, the single mutants demonstrated either an increase or no change in the signal. Double mutants were then tested showing that in some cases, the allosteric effect was lost in *p*-ERK 1/2 signaling, suggesting the involvement of these amino acids in the proposed binding site. In addition, these results revealed the complexity of allostery. On the one hand, we observed a reduction in one signaling pathway and yet an increase in the other. This complicated activity and effect of Ec2la on differing signaling pathways highlight that allosteric effects are not always consistent with every pathway. Part of this complexity may lie with the communication between orthosteric and allosteric sites as can be presented through probe dependency, which is observed with Ec2la. Our results demonstrate that in the presence of varying orthosteric ligands, Ec2la exhibits differing allosteric effects in cAMP experiments; in the presence of JWH133, 2-AG and CP, 55940 we see a negative allosteric effect, yet, with HU308 we see a positive allosteric effect. The observed effects of Ec2la seems to be influenced by the specific orthosteric agonist used to study the interaction. Different probes may achieve different binding modes within an orthosteric site which would then communicate differently with an allosteric bound molecule. The intricate pharmacology of Ec2la has been recently studied,^47^ with results suggesting that its effects differ depending on the assay used with both the CB_1_ and CB_2_ receptors. These results further solidify our findings that Ec2la may alter signaling pathways asymmetrically depending on the orthosteric agonist used and the signaling pathway being studied. Our studies provide crucial mutagenesis and functional data to verify the identified site and in turn provide important knowledge for the subsequent discovery of selective CB_2_ AMs.

## Methods

### Identification of a Putative CB_2_ Allosteric Site and Molecular Docking

All computational studies were performed on the inactive-state human CB_2_ crystal structure (PDB ID:5ZTY)^31^ within the Schrödinger Suite (version 2022-4).^35^ Prior to any computational studies performed, the protein was prepared using the Protein Preparation Workflow^48^ tool with the default settings. All mutated residues were mutated back to their WT ones (L153^4.45^ to T, L78^2.48^ to G, A127^3.46^ to T, E242^6.32^ to R and E304^8.48^ to G), and all ligands and solvents were deleted. The prepared protein was then used for the prediction of putative allosteric binding sites by using SiteMap.^32,34^ For the molecular docking studies, all ligands were imported via SMILES or drawn using the 2D Sketcher and prepared using the ligand preparation workflow, LigPrep,^49^ with default settings. All of the specified chiralities were retained. The receptor grid was generated based on SM2 by using the Receptor Grid Generation tool with default settings.^50^ After generating the receptor grid, GLIDE^51^ in extra-precision (XP) mode was used for the docking calculations given the small docking library size. Evaluation of the generated top-scored ligands was based on the docking score (Table S1) and visual inspection (binding pocket complementarity, projection of lipophilic groups toward the membrane bilayer, and non minimized-resolved intramolecular clashes).

### Site-Directed Mutagenesis of Crucial Amino Acid Residues

Primers were designed for the following mutations: F72^2.42^A, L125^3.44^A, L126^3.45^A, I129^3.48^A, L133^3.52^A, T153^4.45^A, I156^4.48^A, M157^4.49^A, L160^4.52^A, F197^5.46^A, and L201^5.50^A (Table S3). Polymerase chain reactions (PCR) using the Bio-Rad T100TM Thermal Cycler and Applied Biosystems Veriti 96 Well Thermal Cycler to introduce the desired mutations within 3× HA-CB_2_ (cDNA). A reaction mix (25 μL) contained 25 ng DNA template, 1× Phusion HF Buffer (Thermo Fisher Scientific), 200 μM dNTP Mix (Fisher Scientific, 0.5 μM primer pairs and 0.02 U/μL Phusion High-Fidelity DNA Polymerase (2 U/μL). A 3-step protocol for each PCR was performed using conditions suggested by the manufacturer of Phusion DNA polymerase. Restriction enzyme DpnI (2%) (New England Biolab) was added to tubes containing the amplified PCR product. These tubes were placed in a stationary incubator (37 °C, 12–16 h). Bacterial transformation using *E. coli* DH5α competent cells was performed, and the DNA was amplified using the manufacturer’s instructions (Invitrogen PureLink HiPure Plasmid Maxiprep Kit, Fisher Scientific).

### Transient Transfection of CB_2_ Plasmids

HEK293 (ATCC CRL-1573) adherent cells were passaged using Dulbecco’s Modified Eagle’s Medium (DMEM)-high glucose (Sigma-Aldrich). This DMEM was supplemented with penicillin (100 U/mL), streptomycin (100 μg/mL), and heat inactivated fetal bovine serum (FBS, 10%) (PAN-Biotech). Once the HEK293 cells had reached approximately 80% confluency, they were transiently transfected following the reverse transfection method using Lipofectamine 3000 (Thermo Fisher) and the receptor (100 ng/well). Diluted DNA mix was added dropwise to the Lipofectamine mix and incubated for 15 min. The transfection mix (50 μL/well) was added to a white clear bottom 96-well plate (Greiner Bio-One) coated with poly-d-lysine (Sigma-Aldrich) followed by HEK293 cell suspension (7.5 × 10^4^ cells/100 μL). The plate was then incubated (5% CO_2_ atmosphere, 37 °C, 24 h) prior to performing cell signaling assays.

### pGlo-Sensor-22F Biosensor Intracellular cAMP Accumulation Assay

After 24 h of incubation, the transfected HEK293 cells were then subject to starvation (2 h) with serum-free DMEM (100 μL/well). The 96-well plate was then incubated (5% CO_2_ atmosphere, 37 °C, 2 h). Cells were equilibrated in cAMP Buffer (1× HBSS, 24 mM HEPES, 0.1% (w/v) BSA, 3.96 mM NaHCO_3_, 1 mM MgSO_4_, and 1.3 mM CaCl_2_·2H_2_O), supplemented with firefly d-luciferin free acid (0.45 mg/mL) (NanoLight Technology). 80 μL of cAMP buffer was used where both allosteric modulator and agonist were added to the wells, and 90 μL of cAMP buffer was used where just agonist was added to the wells. The 96-well plate was then incubated (28 °C, 1 h). Allosteric modulator was incubated for 20 min prior to measurement. Bioluminescence was then measured using the CLARIOstar Plus Plate Reader (BMG LabTech, Germany). Prior to injecting the treatment ligands, approximately 5–10 basal readings were performed until stabilization was reached. Bioluminescence was measured for a total of 45 cycles (1 min per cycle, 1 s integration time, without filter, a fixed gain of 3000 and autofocus).

### AlphaLISA SureFire Ultra *p*-ERK 1/2 (Thr202/Thr204) Assay

After 24 h of incubation, the transfected HEK293 cells were then subject to starvation (2 h) with serum-free DMEM (200 μL/well). The 96-well plate was then incubated (5% CO_2_ atmosphere, 37 °C, 3 h). Culture media were removed and replaced with allosteric modulator (45 μL/well) prepared in serum-free DMEM and incubated (RT, 30 min). The cells were then stimulated with agonist (5 μL/well) prepared in serum-free DMEM and incubated (RT, 10 min). All subsequent reagents were used from the AlphaLISA SureFire Ultra *p*-ERK 1/2 (Thr202/Tyr204) Assay Kit (PerkinElmer). The medium was removed, freshly prepared 1× lysis buffer (50 μL/well) was added, and the plate was agitated on a plate shaker (RT, 10 min, 350 rpm). The lysates (10 μL) were transferred to a 384-well OptiPlate (PerkinElmer), and the acceptor mix (5 μL) was added to each well under subdued light. The plate was sealed, covered with foil, and incubated (RT, 1 h). Donor mix (5 μL) was added to each well under subdued light. The plate was sealed, covered with foil, and incubated in the dark (RT, 1 h). The plate was read using CLARIOstar Plus Plate Reader (BMG LabTech, Germany) using standard AlphaLISA settings with an emission filter (680–640 nm) and excitation filter (615–618 nm).

### Western Blotting

HEK293 cells were seeded (1,000,000/well) on a 6 well plate coated with poly-d-lysine and incubated (5% CO_2_ atmosphere, 37 °C, 24 h). After incubation, CB_2_ plasmids were transfected into the HEK293 cells using the forward transfection method using polyethyleneimine (Polysciences) and incubated (5% CO_2_ atmosphere, 37 °C, 24 h). After incubation, lysates were collected from the supernatants and quantified using the Bradford assay. The lysate samples (60 μg) were then prepared for the western blot using 6× Laemmli SDS sample buffer (Alfa Aesar) and RIPA buffer. The samples were dispensed into a 15-well Mini Protein Gel (Invitrogen, 4–12%, Bis-Tris, 1.0 mm) and run at 120 V for 90 min. The gel was transferred to a semidry transfer machine (15 V, 1 h). The membrane was added to a 50 mL falcon with blocking buffer (5% skimmed milk powder in TBS-Tween, 5 mL, 1 h, RT). The blocking buffer was then removed, and 1:1000 dilution of primary antibody (HA-tag Rabbit mAb, Cell Signaling Technology, C29F4) and loading dye (mouse β-actin antibody, Abcam, ab8226) were added to blocking buffer and added to the membrane and placed on a roller (4 °C, overnight). The primary antibody was then removed from the tube and washed with TBS-Tween (3 times, 5 min, RT). 1:1000 dilution of secondary antibodies (IRDye 800CW Goat anti-Mouse, C50113-06; Li-COR; IRDye 680RD Goat anti-Rabbit, Li-COR, C50317-02) were added to blocking buffer and then added to the membrane for 1 h, RT. The antibody was removed and washed with TBS-Tween (3 times, 5 min, RT). The membrane was observed using the Li-Cor Odyssey Imaging System.

### In-Cell Western

HEK293 cells were transfected using the reverse transfection methods (described above). Formalin (10%, 150 μL/well) was added for 10 min and incubated at RT and again for 5 min at RT. Wells were washed with phosphate buffered saline (PBS) (3× ∼ 100 μL/well). PBS (100 μL/well) was added to all wells that are nonpermeabilized, and permeabilization buffer (PBS + 1% TWEEN 20, 100 μL/well) was added to the appropriate wells and incubated for 10 min at RT. The contents of all wells were removed, and blocking buffer (1.5 g BSA in 50 mL PBS, 100 μL/well) was added to all wells and incubated for 1 h at RT. The contents of all wells were removed, and the primary antibody (Anti-HA Rabbit, Sigma-Aldrich, H6908) (1:1000, 50 μL/well) was added to appropriate wells and incubated for 1 h at RT. The secondary antibody (Alexa Fluor 488 Goat Anti-Rabbit, Invitrogen, A-11008) (1:1000, 50 μL/well) was added to appropriate wells and incubated for 1 h at RT. The contents of all wells were removed and washed with PBS (3× 100 μL/well). DAPI (1:1000, 50 μL/well) was added to all wells. The contents of all wells were removed and washed with PBS (3× 100 μL/well). PBS (100 μL/well) was then added, and the fluorescence was then measured using the CLARIOstar Plus Plate Reader (BMG LabTech, Germany).

### Data Analyses

Data from signaling experiments were analyzed using GraphPad Prism, version 9.4.1 software (GraphPad Software, Inc.). Western blot quantification analysis was performed using ImageJ. Compounds were drawn using ChemDraw. All computational modeling was performed using tools available within the Schrödinger Suite (2022-4). Sigmoidal concentration response curves were generated by performing AUC analysis. The curves were fit using three-parameter nonlinear regression curves. Statistical analyses were performed on the three independent biological replicates using repeated measures one-way ANOVA with Dunnett’s multiple comparisons tests (* < 0.05) to elucidate the significance of the pIC_50_/pEC_50_ values.

## Supporting Information Available

Top docking scores computed for known CB_2_ allosteric modulators, CBD and Ec2la; top-scored ligands from docking in the putative CB_2_ allosteric binding site; ligand-binding residues between CB_2_ allosteric modulators, CBD and Ec2la, and CB_2_ allosteric site; effect of Ec2la on CB_2_ in the presence of different CB_2_ agonists in cAMP accumulation assays; effect of Ec2la at nanomolar concentrations on CB_2_ in the presence of different CB_2_ agonists in cAMP accumulation assays; forward and reverse primers for CB_2_ mutants; pEC_50_/pIC_50_ values showing the effects of Ec2la on CB_2_ mutants in the presence of CB_2_ agonist JWH133 in cAMP accumulation assays; protein quantification of WT CB_2_ and CB_2_ mutants; and pEC_50_/pIC_50_ values showing the effects of CBD on CB_2_ mutants in the presence of CB_2_ agonist JWH133 in cAMP accumulation assays (PDF)

## Abbreviations

GPCR: G protein-coupled receptor
TM: transmembrane
CB_1_: cannabinoid receptor 1
CB_2_: cannabinoid receptor 2
CBs: cannabinoid receptors
AM: allosteric modulators
PAM: positive allosteric modulator
NAM: negative allosteric modulator
2-AG: 2-arachidonoylglycerol
CBD: cannabidiol
SM1: SiteMap1
SM2: SiteMap2
RMSD: root mean standard deviation
cAMP: cyclic adenosine monophosphate
HEK293: human embryonic kidney cells
*p*-ERK: phosphorylated-extracellular signal-related kinase
PBS: phosphate buffered saline
PCR: polymerase chain reaction
