# Supplementary material for: Identification of a Cannabinoid Receptor 2 Allosteric Site Using Computational Modeling and Pharmacological Analysis
Source: ACS Pharmacol Transl Sci. 2025 Jan 28;8(2):423–34. doi: 10.1021/acsptsci.4c00547 (PMC11833715; doi:10.1021/acsptsci.4c00547)
Supplement: Supplementary file 1 — pt4c00547_si_001.pdf [file pt4c00547_si_001.pdf]

Supporting Information

Identification of a Cannabinoid Receptor 2 Allosteric Site Using Computational Modelling and Pharmacological Analysis

Zara Farooq[1,4], Pietro Delre[2], Stylianos Iliadis[1], Giuseppe Felice Mangiatordi[2], Marialessandra Contino[3], Lesley A. Howell[4] and Peter J. McCormick,[1,5,6]*

[1]Centre for Endocrinology, William Harvey Research Institute, Bart's and The London School of Medicine and Dentistry, Queen Mary University of London, Charterhouse Square, London, EC1M 6BQ, United Kingdom; [2]CNR–Institute of Crystallography, Via Amendola 122/o, 70126 Bari, Italy; [3]Department of Pharmacy-Drug Sciences, University of Bari Aldo Moro, Via Orabona 4, 70125, Bari, Italy; [4]School of Physical and Chemical Sciences, Queen Mary University of London, Mile End Road, London, E1 4NS, United Kingdom; [5]Department of Pharmacology and Therapeutics, Institute of Systems Integrative and Molecular Biology, University of Liverpool, Liverpool, L69 7BE, United Kingdom. [6] XJTLU-University of Liverpool Joint Centre for Pharmacology and Therapeutics.

*Email: peter.mccormick@liverpool.ac.uk

Ligand	Glide Docking Score (kcal/mol)
**CBD**	-5.416
**Ec2la**	-4.439

Table S1 – Top docking scores computed for known CB_2 allosteric modulators, CBD and Ec2la.

Allosteric modulators are listed in descending order after all docking calculations were performed. Once docking calculations were completed, Glide yielded a docking score suggesting that CBD is the best scored ligand. The more negative the values, the stronger the binding.

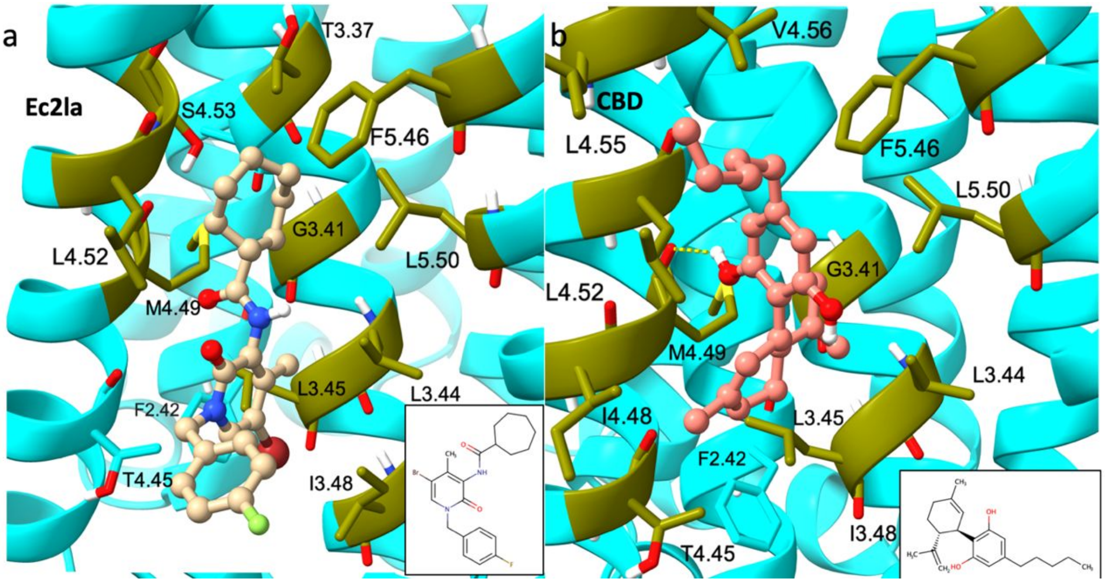

Figure S1 – Top-scored ligands from docking in the putative CB_2 allosteric binding site.

Top-scored docked poses for a) Ec2la (beige) and b) Cannabidiol (CBD) (salmon). CB_2 inactive-state crystal structure (PDB ID: 5ZTY)[31] in shown in cyan. Oxygen atoms in red, nitrogen's in blue and hydrogens in white. Contacts and interacting residues as defined in UCSF ChimeraX[160] are shown with olive carbon atoms. Hydrogen bonding interactions are shown with yellow dashed lines. Ligands are shown in ball-and-stick and residues in stick representation. Images were generated using UCSF ChimeraX.[38]

Allosteric Modulator	Ligand-Binding Residues
**CBD**	G122$^{3.41}$, L125$^{3.44}$, L126$^{3.45}$, I129$^{3.48}$, T153$^{4.45}$, I156$^{4.48}$, M157$^{4.49}$, L160$^{4.52}$, F197$^{5.46}$, L201$^{5.50}$
**Ec2la**	F72$^{2.42}$, L125$^{3.44}$, L126$^{3.45}$, I129$^{3.48}$, I129$^{3.48}$, L133$^{3.52}$, T153$^{4.45}$, I156$^{4.48}$, M157$^{4.49}$, L160$^{4.52}$, F197$^{5.46}$, L201$^{5.50}$

Table S2 – Ligand-binding residues between CB_2 allosteric modulators, CBD and Ec2la, and CB_2 putative allosteric site.

Ligand binding residues of top-scored binding poses from docking of known CB_2 allosteric modulators onto SM2 within the CB_2 protein. All interactions are hydrophobic interactions, except for CBD, where a hydrogen bond is additionally formed with the backbone oxygen of M157$^{4.49}$. The two AMs shared 9 ligand-binding amino acid residues; L125$^{3.44}$, L126$^{3.45}$, I129$^{3.48}$, T153$^{4.45}$, I156$^{4.48}$, M157$^{4.49}$, L160$^{4.52}$, F197$^{5.46}$, L201$^{5.50}$.

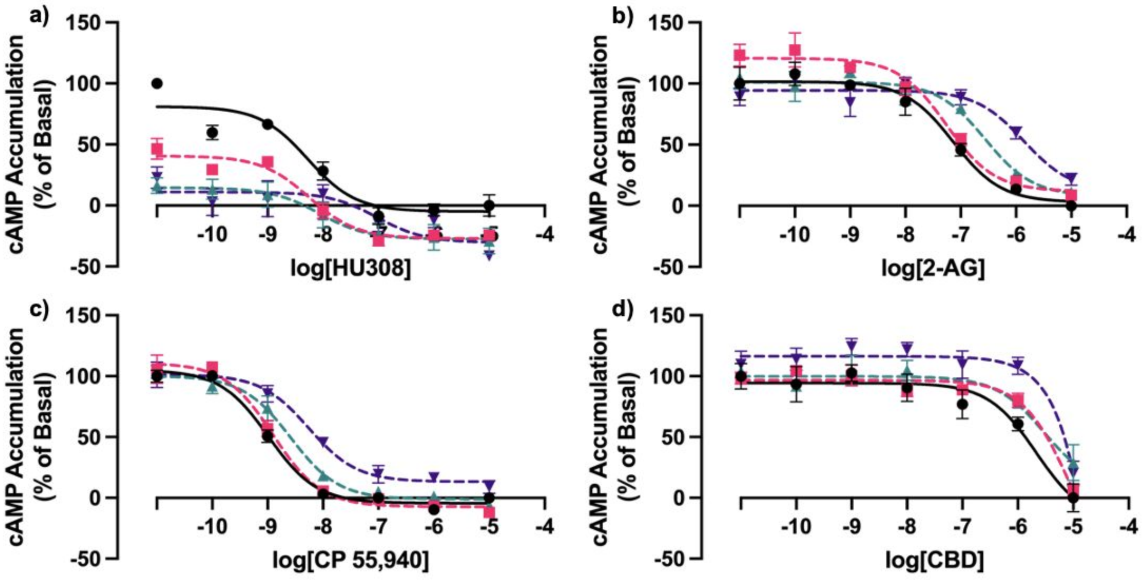

Figure S2 – Effect of Ec2la on CB_2 in the presence of different CB_2 agonists in cAMP accumulation assays.

In FK-induced (7.5 μM) cAMP accumulation dose-response curves for CB_2 using a) HU308, Ec2la demonstrated a possible PAM-antagonistic effect (pEC_{50}/pIC_{50} values; 8.276 ± 0.9, 8.254 ± 0.6, 8.068 ± 1.9, 7.027); vehicle control (no Ec2la), 0.1 μM, 1 μM and 10 μM, respectively), b) 2-AG, Ec2la demonstrated a negative allosteric effect at 1 μM and 10 μM (pEC_{50}/pIC_{50} values; 7.316 ± 0.7, 7.259 ± 0.6, 6.558 ± 0.6, 5.860 ± 1.1*; vehicle control (no Ec2la), 0.1 μM, 1 μM and 10 μM, respectively), c) CP 55,940, Ec2la demonstrated a slight negative allosteric effect at 1 μM and 10 μM (pEC_{50}/pIC_{50} values; 8.985 ± 0.03, 8.903 ± 0.3, 8.585 ± 0.4, 8.258 ± 0.5; vehicle control (no Ec2la), 0.1 μM, 1 μM and 10 μM, respectively), and d) CBD, where Ec2la decreases the potency of Ec2la but the 95% confidence interval values for the EC_{50} are not obtainable (pEC_{50}/pIC_{50} values; 5.671 ± 1.7, 5.045, 5.531, 2.998; vehicle control (no Ec2la), 0.1 μM, 1 μM and 10 μM, respectively). (Black = vehicle control (no Ec2la), pink = 0.1 μM Ec2la, green = 1 μM Ec2la, purple = 10 μM Ec2la). FK alone represents 100%. Data is represented as mean ± SEM as percentage of accumulation normalised to the vehicle control from three independent experiments done in triplicate. Statistical tests to compare the pEC_{50}/pIC_{50} values of each condition of Ec2la (0.1 μM, 1 μM, 10 μM) vs vehicle control (no Ec2la) was performed in GraphPad Prism using a repeated measures one-way ANOVA with Dunnett's multiple comparisons test (* < 0.05).*

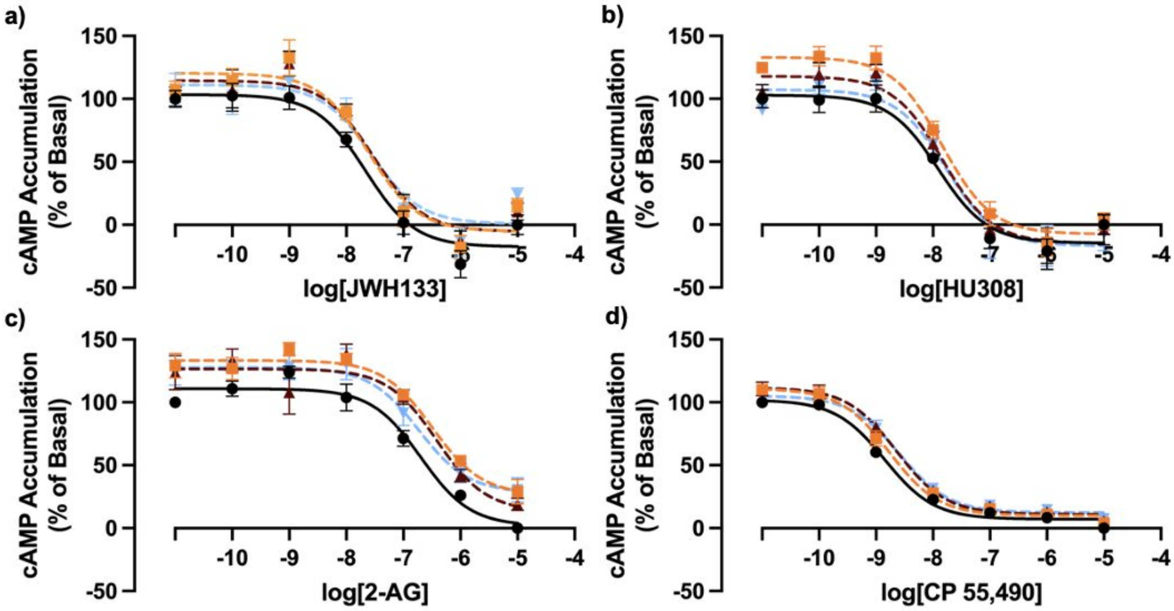

Figure S3 – Effect of Ec2la at nanomolar concentrations on CB_2 in the presence of different CB_2 agonists in cAMP accumulation assays.

In FK-induced (7.5 μM) cAMP accumulation dose-response curves for CB_2, Ec2la did not demonstrate an allosteric effect at nanomolar concentrations using, a) agonist JWH133 (pEC_{50} /pIC_{50} values; 7.665 ± 0.6, 7.613 ± 0.6, 7.531 ± 0.6, 7.566 ± 0.7; vehicle control (no Ec2la), 10 nM, 30 nM and 100 nM, respectively), b) agonist HU308 (pEC_{50}/pIC_{50} values; 7.907 ± 0.5, 7.836 ± 0.4, 7.834 ± 0.6, 7.780 ± 0.6); c) agonist 2-AG (pEC_{50} /pIC_{50} values; 6.694 ± 0.5, 6.493 ± 0.6, 6.417 ± 0.8, 6.731 ± 0.8; vehicle control (no Ec2la), 10 nM, 30 nM and 100 nM, respectively) and d) agonist CP 55,940 (pEC_{50} /pIC_{50} values; 8.849 ± 0.2, 8.769 ± 0.3, 8.643 ± 0.3, 8.584 ± 0.3; vehicle control (no Ec2la), 10 nM, 30 nM and 100 nM, respectively); vehicle control (no Ec2la), 10 nM, 30 nM and 100 nM, respectively). (Black = vehicle control (no Ec2la), orange = 10 nM Ec2la, dark red = 30 nM Ec2la, light blue = 100 nM Ec2la). FK alone represents 100%. Data is represented as mean ± SEM as percentage of accumulation normalised to the vehicle control from three independent experiments done in triplicate. Statistical tests to compare the pEC_{50} /pIC_{50} values of each condition of Ec2la (0.1 μM, 1 μM, 10 μM) vs vehicle control (no Ec2la) was performed in GraphPad Prism using a repeated measures one-way ANOVA with Dunnett's multiple comparisons test (< 0.05).*

Mutation	Primer Design
**M157$^{4.49}$A**	CATCGCGTGGGTCCTCTCAGCAC<br>CCACTGGGACCCGTAGCCGACC
**I156$^{4.48}$A**	GGCGCCATGTGGGTCCTCTCAGCACTAG<br>CGTGACCACTGGGACCCGCGGTACAC
**T153$^{4.45}$A**	GTGGCCCTGGGCATCATGTGG<br>CTTCCCGTGACCACCGGGCA
**I129$^{3.48}$A**	GCCGCTGACCGATACCTCTGCCTGC<br>GGAGGACGACTGGCGGCGACTGG
**L126$^{3.45}$A**	CTGGCGACCGCCATTGACCGATACCTC<br>GACACCCATCGGAGGACCGCTGGC
**L125$^{3.44}$A**	CTCGCGCTGACCGCCATTGACCGATAC<br>CGGAGACACCCATCGGAGCGCGACTG
**F72$^{2.42}$ A**	CATACCTGGCCATTGGCAGCTTGGC<br>GCCTTCGGGAGTATGGACCGGTAAC
**L133$^{3.52}$A**	ATACGCCTGCCTGCGCTATCCAC<br>GGCGGTAACTGGCTATGCGGACG
**F197$^{5.46}$A**	CTGGCCATCGCCTTCCTCTTTTCCGGAATC<br>GACGACTCGACCGAGGACCGGTAGCG
**L201$^{5.50}$A**	CTTCGCCTTTTCCGGAATCATCTACAC<br>GAGGACAAGTGACGGAAGCGGAAAAG
**L160$^{4.52}$A**	GGGTCGCCTCAGCACTAGTCTCCT<br>CCCGTAGTACACCCAGCGGAG

Table S3 – Forward and Reverse Primers for CB_2 mutants.

The method combines primers with 5' complementary sequences containing the point mutations, but with extended non-overlapping 3' ends. The newly synthesised DNA is not 'nicked' allowing it to be used in subsequent amplification cycles which in turn increases the reaction efficiency. All single point mutations were made on the template pcDNA3.1-CB_2.

CB_2 Variant	pEC_{50}/pIC_{50}			
	Vehicle (0 μM Ec2la)	0.1 μM Ec2la	1 μM Ec2la	10 μM Ec2la
**WT CB_2**	8.039 ± 0.4	8.047 ± 0.4	7.333 ± 0.5	6.449 ± 1.7*
**M157A**	7.055 ± 0.8	9.118	8.789 ± 1.9	7.266
**I129A**	8.796 ± 1.5	7.836 ± 1.8	7.711 ± 1.8	7.849 ± 3.0
**L126A**	6.653 ± 1.4	7.147 ± 1.0	6.664 ± 1.8	6.521 ± 2.4
**F197A**	7.951 ± 0.5	7.664 ± 1.0	7.946 ± 2.0	7.835 ± 2.0
**F72A**	7.250 ± 1.2	7.627 ± 1.3	7.552 ± 1.4	7.673 ± 2.7
**L160A**	7.803 ± 0.7	7.797 ± 0.9	7.201 ± 0.8	7.341 ± 1.5*
**L133A**	8.351 ± 1.2	8.714 ± 2.6	7.466 ± 1.5	8.785 ± 1.2
**I156A**	7.775 ± 0.6	7.457 ± 0.6	7.013 ± 0.6*	6.949 ± 2.4*
**T153A**	7.926 ± 0.8	7.700 ± 0.5	7.011 ± 0.9	6.802 ± 1.8*
**L201A**	8.177 ± 1.1	7.551 ± 1.2	7.459 ± 1.4	6.953 ± 1.4*
**L125A**	7.614 ± 0.6	7.433 ± 0.8	6.721 ± 0.5	6.266 ± 1.2*

Table S4 – pEC_{50} /pIC_{50} values showing the effects of Ec2la on CB_2 mutants in the presence of CB_2 agonist JWH133 in cAMP accumulation assays.

pEC_{50} /pIC_{50} values were generated after fitting the curves using three-parameter nonlinear regression. Statistical tests to compare the pEC_{50} /pIC_{50} values of each condition of Ec2la (0.1 μM, 1 μM, 10 μM) vs vehicle control (no Ec2la) was performed in GraphPad Prism using a repeated measures one-way ANOVA with Dunnett's multiple comparisons test (< 0.05).*

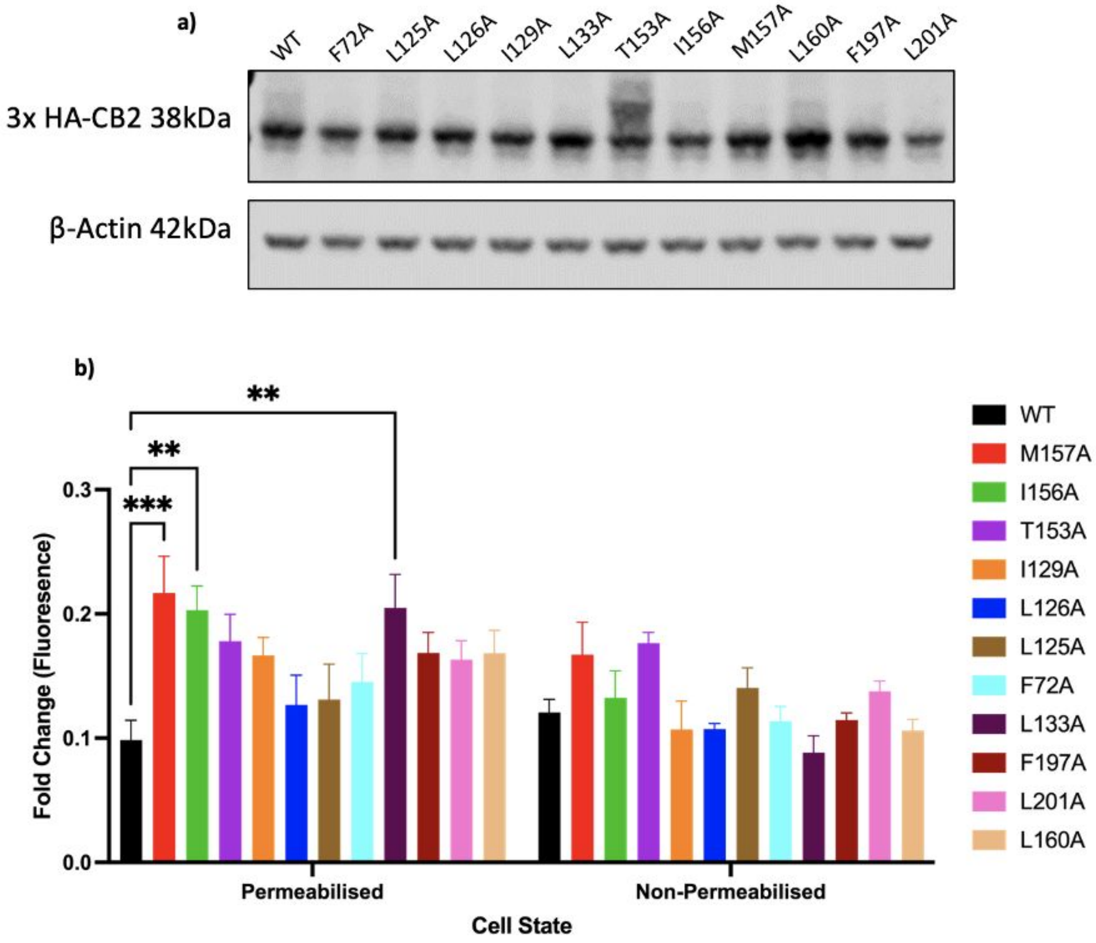

Figure S4 - Protein quantification of WT CB_2 and CB_2 mutants.

a) Western blot of WT CB_2, all CB_2 single mutants and β-actin as loading control. All CB_2 variants are expressing similar amounts of protein, with $L201^{5.50}A$ and $I156^{4.48}A$ showing reduced expression. Data is represented from two independent experiments done in triplicate. b) In-cell western demonstrating that there was no significant difference between the WT-permeabilised cells compared to the mutant permeabilised cells and mutants non-permeabilised cells, apart from $M157^{4.49}A$, $I156^{4.48}A$ and $L133^{3.52}A$ permeabilised cells. Fluorescence values for CB_2 (488-15/535-30) were divided by the DAPI (360-20/460-30). Experiments were done using CB_2-transfected HEK293 cells. Data is represented ± SEM from two independent experiments done in triplicate. Statistical tests were performed in GraphPad Prism using two way ANOVA with Šídák's multiple comparison tests comparing each condition to WT permeabilised.

CB_2 Variant	pEC_{50}/pIC_{50}			
	Vehicle (0 μM Ec2la)	0.1 μM Ec2la	1 μM Ec2la	10 μM Ec2la
WT CB_2	8.030 ± 0.7	7.943 ± 0.7	7.888 ± 0.7	6.994 ± 0.6*
M157A	6.010 ± 2	6.646 ± 2.9	5.748	5.284
I129A	7.387 ± 1.6	7.529 ± 1.1	6.733 ± 1.6	7.341
L126A	7.687 ± 0.6	7.851 ± 1.5	7.684 ± 1.2	7.681 ± 2.4
F197A	8.022 ± 1.1	8.044 ± 1.4	7.490 ± 1.3	7.285 ± 2.4
L160A	7.577 ± 0.7	7.468 ± 0.7	7.367 ± 0.8	6.489 ± 0.3
I156A	7.932 ± 0.4	7.925 ± 0.6	7.740 ± 0.5	7.164 ± 0.9*
T153A	8.035 ± 0.3	7.965 ± 0.4	7.733 ± 0.3	7.147 ± 0.4*
L201A	7.956 ± 0.6	7.335 ± 1.0	7.684 ± 0.6	6.873 ± 0.9
L125A	7.696 ± 0.4	7.852 ± 0.4	7.471 ± 0.6	7.092 ± 1.8*

Table S5 – pEC_{50} /pIC_{50} values showing the effects of CBD on CB_2 mutants in the presence of CB_2 agonist JWH133 in cAMP accumulation assays.

pEC_{50} /pIC_{50} values were generated after fitting the curves using three-parameter nonlinear regression. Statistical tests to compare the pEC_{50} /pIC_{50} values of each condition of CBD (0.1 μM, 1 μM, 10 μM) vs vehicle control (no Ec2la) was performed in GraphPad Prism using a repeated measures one-way ANOVA with Dunnett's multiple comparisons test (< 0.05).*
